# The Staged Photocatalytic Reactor in the Removal of Acetaminophen: Aspects of Adsorption and Photocatalysis

**DOI:** 10.1002/open.202400052

**Published:** 2024-09-09

**Authors:** Claudia Aguilar, Mayra García, Carlos Montalvo, Francisco Anguebes, Mohamed Abatal, Julia Cerón, Rosa Cerón, Sandra Figueroa, Alejandro Ruiz, Marcela Rangel

**Affiliations:** ^1^ Facultad de Química Universidad Autónoma del Carmen Calle 56, No. 4 Av. Concordia Ciudad del Carmen Campeche 24180 México; ^2^ Facultad de Ingeniería Universidad Autónoma del Carmen Av. Central S/N, Esq. Mundo Maya Ciudad del Carmen 24115 Campeche México

**Keywords:** staged photocatalytic reactor, acetaminophen, adsorption, photocatalysis

## Abstract

The efficiency of a staged photocatalytic reactor prototype was evaluated on a semi‐pilot scale with the removal of acetaminophen, for which anatase particles were synthesized by Sol‐Gel and impregnated on rectangular plates of clay. X‐ray diffraction and Energy Dispersive X‐ray Fluorescence patterns show that the final composite is made up of Al_2_O_3_ (14 %), SiO_2_ (41 %), CaO (3 %) TiO_2_ (34 %), and Fe_2_O_3_ (7 %). The impregnation method favors the dispersion of Anatase on the surface of the adsorbent. TiO_2_‐Anatase/Clay, classified as a macro‐porous solid with H3‐type hysteresis loops by N_2_ physisorption. Adsorption processes are improved when using TiO_2_‐Anatase/Clay compared to using TiO_2_‐Anatase. The external mass transfer has a greater influence on the removal rate. The dimensionless parameters of the Biot number indicate there are no limitations due to the diffusive effect on the interior of the particle. The evaluation of the kinetic data under the Langmuir‐Hinshelwood equation shows a decrease in efficiency as the initial concentration increases. The acetaminophen molecule shows destabilization in the structure of the aromatic ring with a visible decrease in the signals of this functional group evaluated by High‐Performance Liquid Chromatography and Raman Spectroscopy.

## Graphical abstract

A stepped photocatalytic reactor prototype was evaluated on a semi‐pilot scale for the removal of acetaminophen, using anatase particles impregnated on clay. Characterization showed a diverse final composition and improved anatase dispersion. Enhanced external mass transfer and adsorption processes influence the removal efficiency.

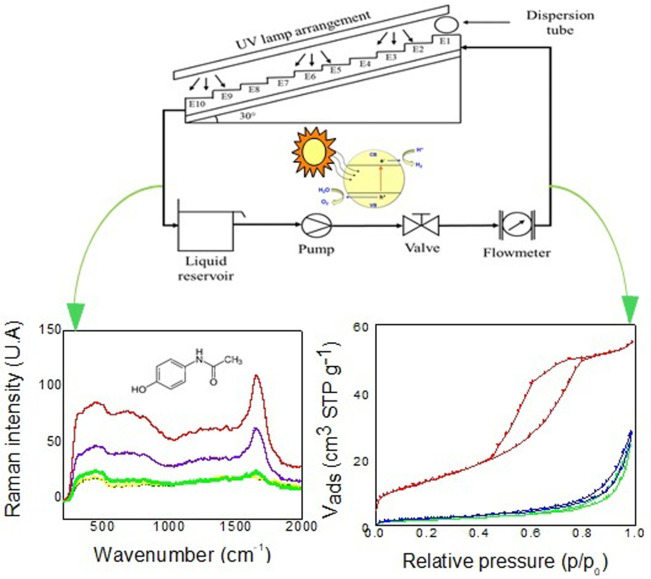

## Introduction

Heterogeneous photocatalysis is an efficient process for the removal of numerous organic compounds, the fundamental principle is based on the use of semiconductor material and radiant energy.^[1]^ For each photon of light absorbed, the excitation of an electron is promoted from the valence band to the conduction band, and the electron‐hole pairs ($e_{cb}^{-}+h_{vb}^{+}$ ) generated during the process lead to the realization of a series of oxidation‐reduction reactions and generation of hydroxyl radicals (•OH) whose objective is to mineralize the organic compound to species such as CO_2_, H_2_O, and inorganic ions.^[2]^

These processes can occur in suspension or immobilized reactors, with various types available (packed bed, annular, suspension, plug flow, falling film photo reactors).^[3]^ Designing optimal contact between photons, photocatalysts, and degradation‐promoting reagents is a key challenge.^[4]^ Factors such as capacity, robustness, reliability, economic feasibility, and practicality must also be considered during scaling.

The *“step”* reactors have the premise of being a technology whose design increases mass transfer (compared to flat and inclined plate designs) and offers a greater surface area with significant degradation percentages of organic compounds.^[5]^ The design and process parameters primarily hinge on reaction kinetics, hydrodynamics, and mass transfer. Among these, energy consumption, particularly through absorption and dispersion by particles, stands out as the most significant design hurdle.^[7]^ The mass transfer rate between the overall fluid and the catalyst surface is a measure of the mass transfer coefficient, which is related to fluid properties, dynamic characteristics, and system geometry.^[8]^

In the case of immobilized bed reactors, the performance is limited by the external mass transfer^[5]^ in certain contact regions the mass transfer between the overall fluid and the surface of the catalyst particle occurs by convection, a mechanism that will dominate as the distance from the surface increases^[9]^ if the flow of the fluid is laminar, then all the transfer between the surface and the moving fluid will be carried out by molecular means if the fluid is turbulent, there will be physical movement along the flow lines resulting in faster processes.^[8]^

The volumetric flow rate influences the external diffusion processes of a catalytic reaction when the solution flows freely over the active surface,^[5]^ the catalyst particles are surrounded by a static layer of solution molecules. The velocity of the solution depends on this layer, increasing the volumetric flow rate improves the diffusion rate, improving the fluid mixing and ensuring that the concentration of reactants and degradation products is approximately the same both at the reaction surface and in the effluent.^[9]^ In this work, the efficiency of a stepped photocatalytic reactor (SPR) prototype with a catalyst synthesized and supported on commercial clay was evaluated, evaluating the effects of adsorption and photocatalytic degradation using models to evaluate the effect of adsorption, mass transfer, and chemical kinetics.

## Results and Discussion

### Characterization of TiO_2_‐Anatase/Clay

#### N_2_ Physisorption

**Figure 1:**
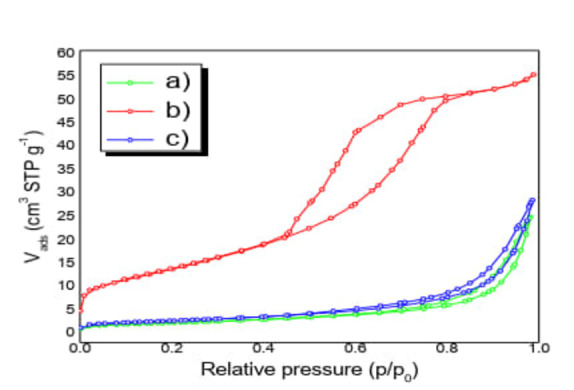
**(a–c)** N_2_ Adsorption‐ Desorption isotherms: a) Clay, b) TiO_2_‐anatase c) TiO_2_‐anatase/Clay.

Figure 1 (a‐c) shows nitrogen adsorption‐desorption isotherms. Classified according to the International Union of Pure and Applied Chemistry (IUPAC criteria): a) for clay, the behavior of the isotherm resembles type II. When the adsorption isotherm does not coincide with the desorption isotherm, hysteresis occurs; for clay, hysteresis loops are classified as type H3 with slit‐shaped pores typical of stratified materials. b) TiO_2_‐anatase follows a behavior similar to a type IV isotherm typical of mesoporous solid materials (predominant pore size between 2 and 50 nm). The hysteresis loop of the TiO_2_ isotherm resembles a distinctive H2‐type one of mesoporous materials with bottle‐type pore necks^[10]^ whose size and pore size distribution are not well defined.

For c) TiO_2_‐anatase/Clay, the data conform to a type III isotherm characteristic of macro‐porous materials (predominant pore size greater than 50 nm), where the monolayer formation is not identifiable and the adsorbed (N_2_) molecules are clustered around the most favorable sites on the surface.,^[11]^ the hysteresis loops are of the H3 type with slit‐shaped pores in layered materials.^[12]^ Table 1 shows the condensed results.

**Table 1:** N_2_ physisorption results under conditions: a) Clay: 300 °C, 60 min b) TiO_2_‐anatase: 250 °C, 180 min c) TiO_2_‐anatase/Clay: 250 °C, 180 min.

Sample	Superficial area (m^2^ g^−1^)	Pore volume (cm^3^ g^−1^)	Pore Diameter (nm)
a)	6.429	0.037	23.445
b)	49	0.085	6.922
c)	8.055	0.043	21.492

The direct impregnation method^[13]^ favors the dispersion of the anatase particles. When the water evaporates, micro‐fractures are produced in the deposits that leave areas of the clay pores available. The migration and deposition of anatase particles towards the interior of the macro and mesopores, distributing them in the laminar structure of the clay cause a decrease in the surface area.

#### X‐Ray Diffraction (XRD)

**Figure 2:**
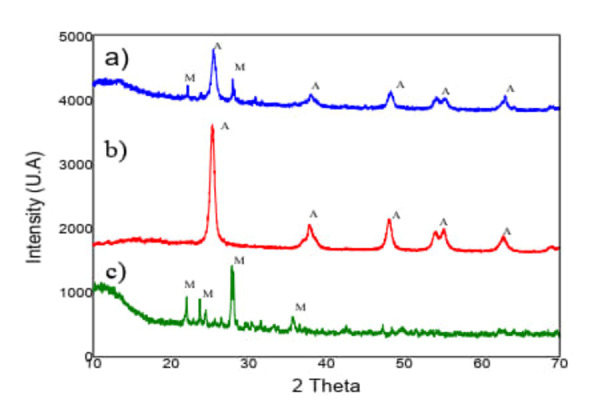
**(a–c)** XRD pattern: a) TiO_2_‐anatase/Clay, b) TiO_2_‐anatase c) Clay (The letters indicate A= Anatase and M=Montmorillonite).

In the XRD pattern of Figure 2‐a) the peaks of the composite formed by clay and TiO_2_‐ anatase are shown, the anatase peaks are identified at 25.12°, 38.55°, 48.15°, 55.16, ° 62.67°, and the peak of the montmorillonite at 22, 28°.

Figure 2‐b) shows a tetragonal structure with characteristic peaks at 25.12°, 38.55°, 48.15°, 55.16°, 62.67° and 68.36°, whose sharpness and intensity are distinctive of TiO_2_ – anatase particles.

Figure 2‐c) shows the clay structure with signals corresponding to montmorillonite, low‐calcium montmorillonite‐type clays are common in Mexican soils, formed through the alteration of silicate minerals under alkaline conditions.

The regulation of the conditions in the development of the synthesis method (precursor, calcination temperature, aging time, agitation) is fundamental in the micro‐structural properties of the TiO_2_‐anatase.[^14^, ^15^]

Its elemental composition gives clay cationic exchange capacities that make it susceptible to supporting TiO_2_ in photocatalysis processes for removing aromatic molecules^[16]^ and antibiotics.^[17]^

TiO_2_‐anatase consists of fine particles prone to agglomeration on support material surfaces. Studies indicate that microfibrous and tubular clay morphologies enhance deposition and dispersion capacity, preventing particle agglomeration and boosting photocatalytic activity.^[18]^ Montmorillonite, classified as a phyllosilicate, features small laminar or fibrous crystals that facilitate Anatase dispersion on its surface.

By N_2_ physisorption, it is observed that the TiO_2_‐anatase/Clay composite would have the adsorption capacity; the diameter of its pores increases accordingly to TiO_2_‐anatase and maintains the properties of a photocatalyst, the crystallographic properties are not altered when deposited in the clay, these conditions could increase the efficiency of the photocatalytic reaction.

#### Energy Dispersive X‐Ray Fluorescence (EDXRF)

**Figure 3:**
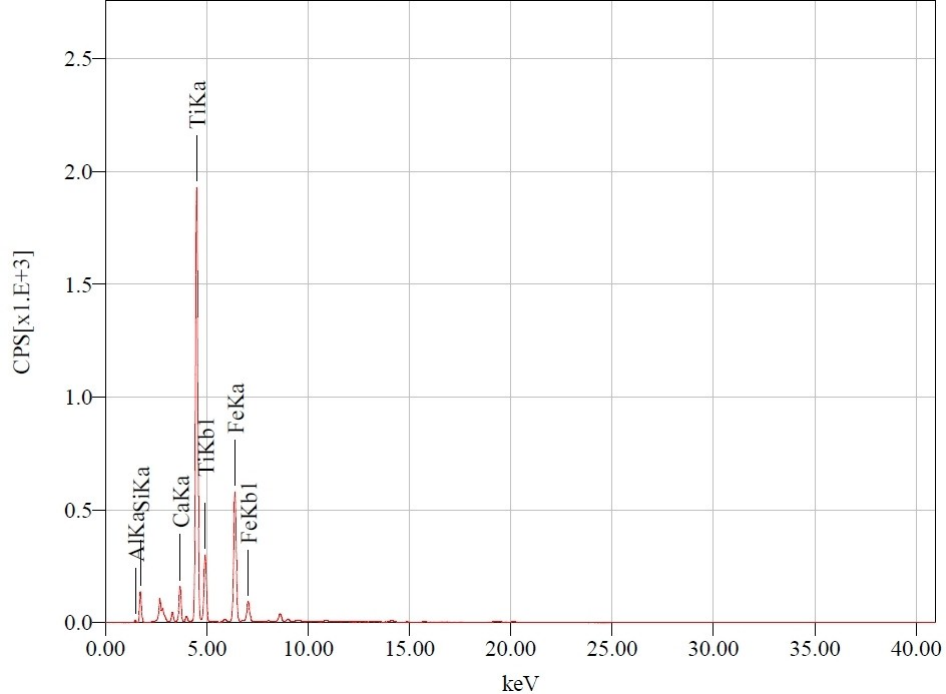
EDXRF pattern of TiO_2_‐anatase/Clay composite the peaks of the main determined oxides are shown.

The elemental composition of TiO_2_‐anatase/Clay by EDXRF (Figure 3) showed that the composite contains Al_2_O_3_ (14 %), SiO_2_ (41 %), CaO (3 %) TiO_2_ (34) and Fe_2_O_3_ (7 %). The acetaminophen (ACET) molecule is a weak acid (pKa=9.38) in aqueous media it dissociates, and part of its concentration is found as phenolates. The adsorption of TiO_2_‐anatase/Clay is enhanced by its basic surface conditions, facilitating acid‐base interactions with acetaminophen‘s resonant structure. Adsorption mechanisms from the aqueous phase may involve the formation of electron donor‐acceptor complexes with amide and phenol functional groups. However, the adsorption capacity decreases with the surface oxidation of TiO_2_‐anatase/Clay.

#### Adsorption

**Figure 4:**
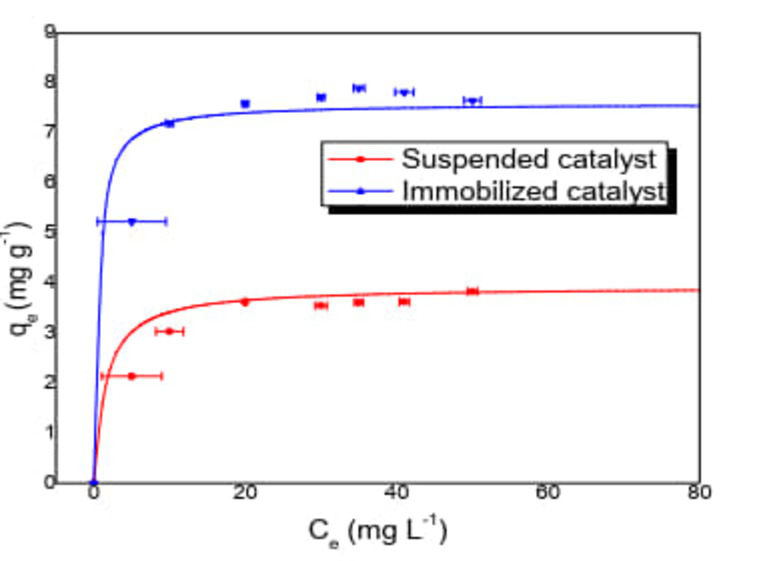
Comparison of the adsorption capacity of TiO_2_‐anatase catalyst in suspension and immobilized TiO_2_‐anatase/Clay (Solid line =model; dots=experimental data).

Adsorption tests were carried out with the catalyst suspended and immobilized on clay particles (mass=1 g), with a 250 rpm agitation, varying the concentration of ACET of 0–50 mg L^−1^, without modifications in the pH value= 6.8. The results are shown in Figure 4, in both cases the adsorption isotherm was adjusted to the classic type I of the Langmuir isotherm according to the classification adopted by the IUPAC,^[19]^ the adsorbent‐absorbate interaction potentials are significant and chemisorption processes show this type of isotherm that is characterized by the formation of a monolayer. The values obtained from the comparison of both processes are: for the immobilized catalyst $q_{m}=7.598\;{mg\;g}^{-1}\;K_{L}=1.9323\;{L\;mg}^{-1}\;R^{2}=0.9986$ ; with a maximum percentage of adsorption of 17.564 %. For the catalyst in suspension $q_{m}=3.420\;{mg\;g}^{-1}\;K_{L}=0.675\;{L\;mg}^{-1}\;R^{2}=0.9921$ ; with a maximum percentage of adsorption of 3.521 %. Equilibrium is reached in 240 min contact time. These results show that the adsorption capacity increases when the catalyst is supported on clay. For the adjustment of the data, the average percentage deviation (% D) was made Where: n is the number of experimental points $q_{ei,exp}$ is the point of experimental adsorption at ${mg\;g}^{-1}\;$ and $q_{ei,cal}$ is the predicted adsorption at each point in ${mg\;g}^{-1}\;$ (Equation 1.
(1)

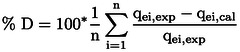




The global deviation percentage for the suspended system is % D= 8.8112 and for the immobilized system %D=11.2823 .

#### Photocatalysis

**Figure 5:**
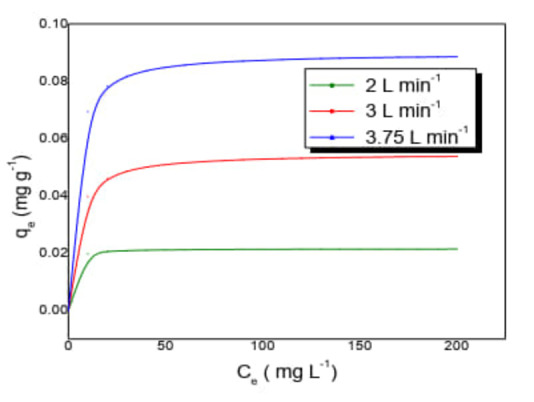
Evaluation of the optimal flow for degradation reactions in the SPR (Irradiation time 520 min, three lamps of λ =365 nm, pH=6.8, mass of immobilized catalyst=24 g).

In photocatalytic processes, the adsorption has a fundamental role,[^20^, ^21^] when the catalyst is supported on an adsorbent. Prior to the photocatalytic reactions, the optimal flow ${(Q}_{optimun})$ in the SPR is estimated, (Figure 5) at three different flow rate conditions 3.75 L min^−1^ being the optimal value. The reactor geometry and the arrangement of the plates with the supported catalyst increase the interfacial area of the catalyst, the stepped arrangement of the plates favors flow conditions between laminar and turbulent, which favors adsorption.

High residence times result in slow flow rates.^[22]^ However, high residence times that do not increase the fluid film thickness are necessary to promote reactor efficiency and this is achieved by increasing the flow velocity.

SPR efficiency is evaluated in different reactions varying the initial concentration of ACET to promote the adsorption on the catalyst surface, the solution was recirculated in the dark phase (Time=120 min), subsequently, the solution was irradiated by 3 UV lamps of λ=365 nm, samples of 5 mL were extracted every 60 min and analyzed by High‐Performance Liquid Chromatography (HPLC) at the end of each reaction. The TiO_2_‐anatase/Clay plates were washed with deionized water and subjected to heat treatment at 200 °C to reverse the adsorption of unwanted organic compounds.

The degradation percentages were evaluated with Equation 2, where $C_{f}$ =final concentration and $C_{0}$ =Initial concentration
(2)

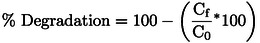




When $C_{0}$ of ACET increases the percentage of degradation decreases considerably (maximum percentage of degradation=74.76, and the minimum=35.74).

The kinetic constants$(K_{1}=621.978\;{min}^{-1}\;K_{2}=1.28375\;{mg\;L}^{-1})\;$ were determined with Langmuir Hinshelwood (LH) equation (3) using optimization by nonlinear regression and the Levenberg‐Marquardt equation with the Statistical 7.1 software.

(3) $${-r}_{A}=-\frac{{dC}_{A}}{d_{t}}=\frac{K_{1}C_{A}}{1+K_{2}C_{A}}$$

**Figure 6:**
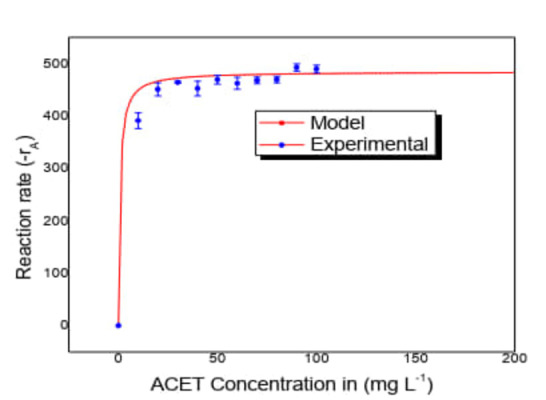
LH model for the evaluation of experimental data at different concentrations of ACET ($Q_{optimun}$ =3.75 L min^−1^, Irradiation time= 520 min, three lamps of λ =365 nm, pH=6.8, mass of immobilized catalyst=24 g).

As in our results, other research has concluded that TiO_2_ impregnated on clay improves the adsorption capacity, and the chemical stability and cation exchange capacity of the support suggest a synergistic effect between clay and the TiO_2_ particles,[^23^, ^24^, ^25^] in which the TiO_2_ agglomerations act as a light absorber, while clay operates as a physical adsorbent of organic molecules, increasing the performance of photocatalytic oxidation processes in aqueous suspensions. The evaluation of the reaction rate as a function of concentration shows that for the LH equation (Figure 6) the trend of the data with increasing concentration is close to zero order at the transition of the curve.

The SPR efficiency of this study compared with a parabolic trough reactor (CPC) with TiO_2_ in suspension,^[26]^ shows that the SPR can be as efficient as the CPC reactor in removal percentages. The energy source can be a determining factor in the efficiency of stepped reactors,^[27]^ factors such as the initial concentration, the flow rate, and the immersion time of the support significantly influence the removal efficiency, In our study, the maximum removal percentages were 74.76 %, higher than those reported in this study.

#### Chemical Degradation Followed by Raman Spectroscopy (RS) and High‐Performance Liquid Chromatography (HPLC).

**Figure 7:**
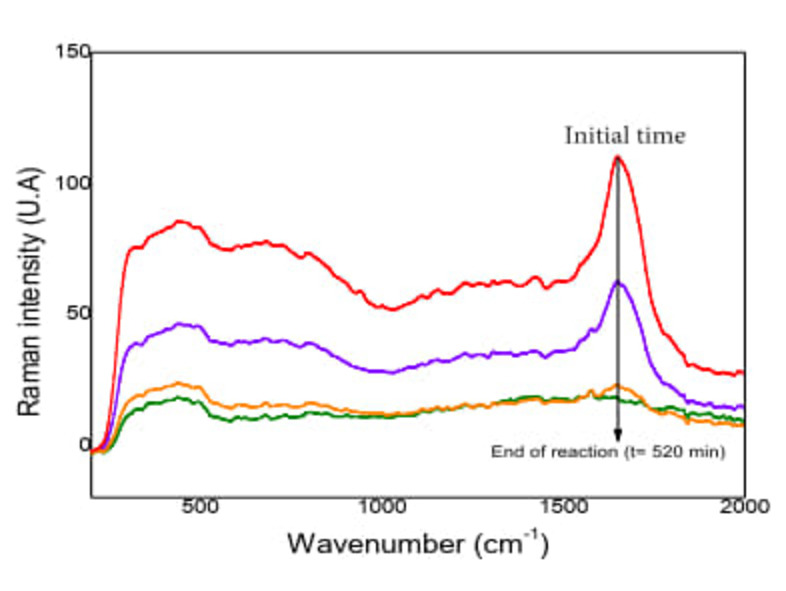
Raman spectra of the photocatalytic degradation of ACET $C_{0}=50\;{mg\;L}^{-1}$ ($Q_{optimun}$ =3.75 L min^−1^, Irradiation time= 520 min, three lamps of λ =365 nm, pH=6.8, mass of immobilized catalyst=24 g).

Figure 7 corresponds to the samples obtained in the degradation of ACET at 50 mg L^−1^, the spectrum of the unreacted molecule is shown well defined highlighting the presence of characteristic peaks,^[28]^ which are summarized in Table 2. In solution, ACET has Raman bands that overlap each other;^[29]^ as the reaction proceeds, the decrease of the peak at 1673 cm^−1^ is shown; corresponding to the stretching of the aromatic ring; evidence of chemical degradation of the molecule.

**Table 2:** Assignment of Functional Groups to the RS

Wavenumber (cm^−1^)	Assignment
872	C−N bond stretching
1186	*P*‐substituted benzene ring.
1254	Aromatic (out of phase stretching C−C−O).
1345	Aromatic (involves bending at C−O−H).
1392	Amide III (C−N bond stretch).
1561	Amide II (Bending in C−N−H).
1673	Aromatic ring stretching

**Figure 8:**
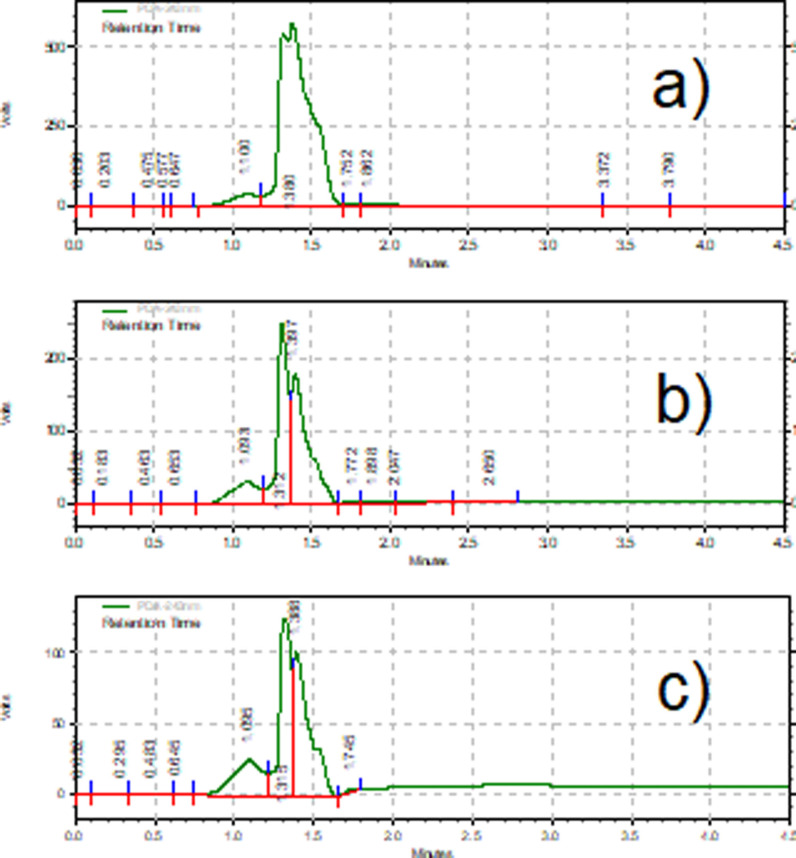
**(a–c)**. Chromatogram of the photocatalytic degradation of ACET at $C_{0}=100\;{mg\;L}^{-1}$ ($Q_{optimun}$ =3.75 L min^−1^, Irradiation time= 520 min, three lamps of λ =365 nm, pH=6.8, mass of immobilized catalyst=24 g).

By HPLC the degradation of the ACET molecule is shown in Figure 8 (a‐c), at the beginning of the reaction (t=0); the characteristic signal of the molecule is identified (Figure 8‐a) with a retention time of 1.380 min; as the reaction proceeds (Figure 8‐b) the instability in the aromatic structure is shown due to possible substitution processes of functional groups, the ( 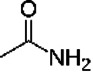) acetamide is a structure susceptible to the attack of the⋅OH radicals in its C−N bonds by hydroxylation ( Figure 8‐c) show that the aromatic signal‘s intensity has decreased, agreeing with the kinetic values.

#### Mass Transfer

In catalytic processes, efficiency is often limited by external and internal diffusion. Reactions happen when reactant molecules diffuse through a fluid layer surrounding a catalyst particle, and then through the pore inside the particle.

In the external film diffusion model, the following assumptions are made: the adsorbent particles are spherical, perfect mixing conditions are present, internal diffusion is not significant during initial adsorption, the adsorbate disappearance rate is equal to the adsorption rate to the outside of the particle.^[30]^

For immobilized bed reactors, performance is limited by external mass transfer, the substrate concentration is different in the solution and on the catalyst surface. Table 3 shows the results of the calculation of K_f_; compared to other studies,^[31]^ in suspended media, our results are lower indicating that the transfer from the fluid to the catalyst surface is slower in an immobilized system. The dimensionless Biot number parameter (Equation 4.

(4) $$\;B_{i}=\frac{k_{f}R}{D_{ef}}$$

**Table 3:** Predicted values for ACET adsorption and K_f_ on TiO_2_‐Anatase/Clay in an SPR at different adsorbate concentrations.

$C_{0}$ (mg L^−1^)	Adsorption (%)	$K_{f}$ (m min^−1^)	$D_{ef}\;\left(m^{2}{min}^{-1}\right)$
20	17.3882	2.1343×10^−3^	4.4117×10^−5^
30	15.7026	1.6458×^−3^	2.3909×10^−5^
40	16.0961	1.6994×10^−3^	1.4572×10^−5^

defines a relative relationship between mass transfer around the particle and mass transfer within the particle expressed by the $D_{ef}$ =Effective diffusivity when the value of $B_{i}$ ⋙100 it is assumed that the resistance to mass transfer is inside the particle.

The parameter $D_{ef}\;$ is used for intermediate pore sizes and assumes that the diffusion paths are tortuous. Internal diffusion $D_{M}$ =Molecular diffusion and was evaluated based on the Wilke Chang Equation, the different viscosities (μ) .

The porosity in TiO_2_‐anatase/Clay ($\epsilon_{p})$ was established with the equation 5.

(5) $${\;\epsilon }_{p}=\;\frac{V_{g}}{V_{p}}=\frac{Pore\;volume}{Particle\;volume}=\;0.4410$$

The tortuosity (τ=12) , for the TiO_2_ ^[31]^ calculated values are shown for $D_{ef}$ (Equation 6.

(6) $$D_{ef}=\frac{D_{M}\epsilon_{p}}{\tau }=\;\frac{{\left(7.4\;x\;10^{-8}\right){\left(\Phi M\right)}^{0.5}T\epsilon }_{p}}{\mu V_{m}^{0.6}\tau }$$

**Figure 9:**
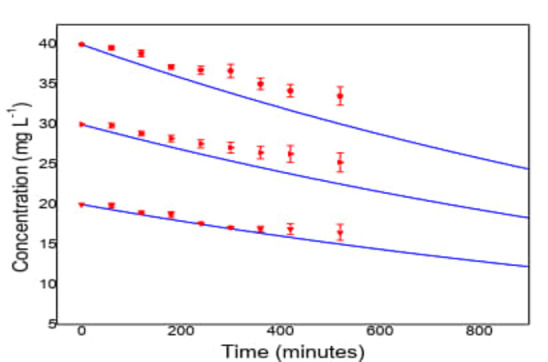
ACET adsorption on TiO_2_‐anatase /Clay (pH= 6.8; C_o_=20‐40 mg L^−1^), the continuous lines represent the model, and the dots the experimental data.

In all cases the Bi number is less than 100; the greatest resistance to mass transfer is not inside the particle, and efficiency is related to external diffusion. In Figure 9; the application of the film and pore model in the adsorption of ACET is shown, and the concentration decay curves are influenced by the adsorption processes.

An ordered structure in the adsorbent material would lead to lower tortuosity values (homogeneous pores) and therefore to higher effective diffusivities; for TiO_2_‐anatase/Clay the formation of conglomerates on the clay surface could partially block the pores and therefore a decrease in the performance in internal diffusion processes, the pore size for TiO_2_‐anatase/Clay did not must be limiting.

## Conclusions

The photocatalytic reaction prototype (SPR) effectively removes acetaminophen. High conversion percentages are achieved, especially at low initial concentrations. There is clear evidence of chemical transformation of the molecule. The structure of the TiO_2_‐anatase/clay; favors acetaminophen adsorption processes by acid‐base interactions forming donor‐acceptor complexes. The adsorption capacity decreases by oxidation on the TiO_2_‐anatase/Clay surface in the photocatalytic process. The volumetric flow rate and reactor geometry promote transitional flows between laminar and turbulent conditions. Internal mass transfer is not limited according to the Biot number, but external mass transfer significantly affects the photocatalysis process. It′s concluded that adsorption contributes to some of the acetaminophen removal, and the radiation isn′t enough to completely mineralize it at high initial concentrations.

## Experimental Section

### Materials and Instruments

ACET used in degradation reactions is from Sigma Aldrich (CAS number 103–90‐2). The catalyst TiO_2_ was synthesized by the Sol‐Gel method using titanium butoxide as a precursor (CAS number 5593–70‐4), and and 1‐butanol (CAS number 71–36‐3).To establish the characteristics of the composite (surface area; diameter and pore volumes), N_2_ Physisorption was used in a Belsorp II mini equipment (Bel Japan). The crystalline phase was determined using X‐ray diffraction (XRD) with a Bruker AXS D8 Advance Diffractometer. Elemental analysis was conducted via Energy dispersive X‐ray fluorescence (EDXRF) using a JEOL, JSX‐1000 s. ACET degradation was assessed by HPLC using an Agilent 1100 system equipped with a quaternary pump and a Zorbax (C‐18) column. The mobile phase, prepared with water (HPLC grade) from Merck and methanol (spectroscopic grade) from Fischer Scientific, was gradient‐based. Prior to analysis, reaction samples were filtered through a 0.22 μm cellulose acetate filter (Millipore Corp., Bedford, MA).

Changes in chemical structure were identified using Raman spectroscopy (RS) with a QE 65000 from Ocean Optics, adapted with an RPB 785 fiber optic probe and spectrograph. The viscosity and density of the ACET solution were measured using an Anton Paar Rheometer Physica MCR 101.

### Synthesis of TiO_2_‐Anatase/Clay

TiO_2_ was synthesized by the Sol‐Gel method using titanium butoxide as a precursor. The procedure is summarized in the following steps^:[32]^ in a glass reactor, titanium butoxide (24.3851 g) was mixed with 3/4 parts of the total mass of 1‐butanol (29.5327 g) under controlled and continuous stirring at 250 rpm in a digital propeller shaker (Model RW 20, IKA). After one hour of reaction, 1/4 part of the remaining alcohol (9.8453 g), as well as deionized water (7.8 g), is added by slow dripping. The mixture remains under reflux and is stirred for two hours at a controlled temperature of 65 °C. Finally, the solid is allowed to settle (age), dried at 120 °C and calcined at 550 °C for 300 min; this process generates a final 5–6 g of TiO_2_, this solid is deposited by direct impregnation,^[13]^ on clay plates previously treated under a thermal process at 400 °C.

### Stepped Photocatalytic Reactor (SPR)

**Figure 10:**
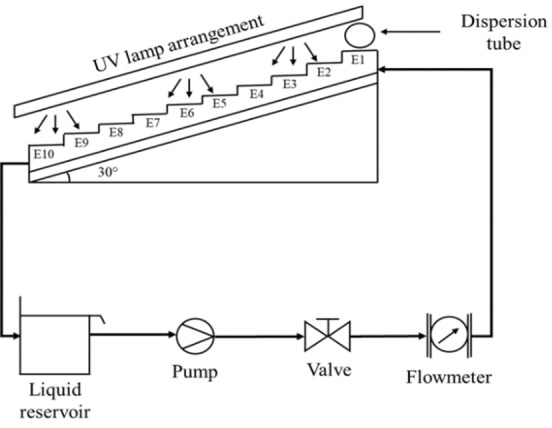
Scheme of the prototype: Stepped Photocatalytic Reactor (SPR), operated continuously (E1‐E10: number of stages).

Figure 10 shows the SPR diagram, the reaction surface consists of commercial clay plates impregnated with the synthesized catalyst and the reaction panel is located at an inclination angle of 30°. A shut‐off valve and a flowmeter are available (Table 4). To determine the optimal reaction flow rate $(Q_{optimun}$ ), tests were carried out with different flows.

**Table 4:** SPR: specifications and materials

Tubular base	Iron
Reactor structure	Stainless steel (26 caliber)
UV Lamps	Three lamps (15 watts, 365 nm) Cole Parmer brand
Clay plates	Dimensions: 37 cm×3.5 cm×2 cm, Area=0.1666 m^2^
Electric pump	Portable diaphragm: 6 L min^−1^, 12v, 70 watts
Flowmeter	Chinwey brand: LZM–15 ( 1–4 L min^−1^)
Liquid reservoir	Volume= 6 L

### Evaluation of the Kinetics of Adsorption and Photocatalytic Reaction.

To determine the adsorption capacity of TiO_2_ in suspension and immobilized, the experimental data were evaluated with the Langmuir Isotherm equation (Equation 7.

(7) $$q_{e}=\frac{q_{m}K_{L}C_{e}}{1+K_{L}C_{e}}$$

To evaluate the global efficiency of the SPR different reactions were carried out varying the initial concentration of ACET, the solution was irradiated by 3 UV lamps of λ=365 nm, with a retention time of TR=520 min, and the total volume of V=4 L and a flow rate of $Q_{optimun}$ =3.75 L min^−1^ samples were analyzed by HPLC at the end of each reaction, the TiO_2_/Clay plates were washed with deionized water and subjected to heat treatment at 200 °C to reverse the adsorption of organic compounds that could interfere in the following reaction.

The LH equation (Equation 3) was used to evaluate the photocatalytic reaction and the obtaining of the kinetic and adsorption constants (K_1_ and K_2_) using nonlinear regression with the Levenberg‐Marquardt equation and Statistical 7.1 software.

### Mass Transfer Effects: External Mass Transfer

To evaluate the external mass transfer, it was assumed that the external film surrounding the solid particle is directly proportional to the concentration difference that exists between the concentration in the solution (C_s_) and the concentration in the solid (C_t_). The value of K_f_ was determined with Equation 8, by nonlinear regression using the Levenberg‐Marquardt equation, and the Statistical 7.1 software. The assumed conditions are $C=C_{0}\;to\;t=0\;and\;C_{s}=0\;to\;t=0$ , V=4 L; At =193.32 m^2^ the total contact area was considered, (24 g= total amount of catalyst mass per surface area 8.055 m^2^ g^−1^). The total area of the impregnated plates =1.66 m^2^

(8)

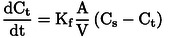




### Internal Mass Transfer: Pore Diffusion

The transport processes that follow Fick's Law in a spherical particle can be expressed by Equation 9 and have been used in pore and film models[^30^, ^33^, ^34^] in adsorption processes and in photocatalysis processes^[31]^ to substantiate the importance of adsorption.

(9) $$\epsilon_{p}\frac{\partial Ci}{\partial t}=D_{ef}\left(\frac{\partial^{2}C_{i}}{{\partial r}^{2}}+\frac{1}{r}\frac{\partial C_{i}}{\partial r}\right)-\rho_{P}\frac{\partial_{qi}}{\partial t}\;$$

(10) $$\mathrm{The}\;\mathrm{initial}\;\mathrm{conditions}\;\mathrm{are}:\;C_{i}=0\;\;t=0$$

(11) $$\mathrm{At}\;\mathrm{the}\;\mathrm{center}\;\mathrm{of}\;\mathrm{the}\;\mathrm{particle}\;r=0\;\frac{{\partial C}_{i}}{\partial r}=0$$

At the surface, the rate at which the solute is transferred through the outer film must be equal to the rate at which it diffuses into the solid, the second boundary condition is:

(12) $$For,\;r=R;\;\frac{{\partial C}_{i}}{\partial r}=\;\frac{k_{f}}{D_{ef}}\left(C_{t}-C_{s}\right)$$

It is assumed that there is a spontaneous equilibrium at the liquid‐solid interface inside the pores, this expression can be related to the Langmuir Isotherm.

(13) $$q_{e}=\frac{q_{m}K_{L}C_{e}}{1+K_{L}C_{e}}\;where\;q_{i}={fC}_{i}\;y\;\frac{\partial qi}{\partial t}=\frac{q_{e}K_{L}}{{\left(1+{K_{L}C}_{i}\right)}^{2}}\;\frac{{\partial fC}_{i}}{\partial t}$$

The concentration drop model of the equation is expressed in Equation 14.

(14) $$\epsilon_{p}\frac{\partial Ci}{\partial t}+\rho_{P}\frac{{\partial fC}_{i}}{\partial t}=D_{ef}\left(\frac{\partial^{2}C_{i}}{{\partial r}^{2}}+\frac{1}{R}\frac{\partial C_{i}}{\partial r}\right)$$

The equation was solved by numerical methods, discretizing the radial derivatives by central finite differences.

## Nomenclature

$Q_{optimun}$
: Optimal flow
$q_{e}$
: The amount adsorbed per weight of adsorbent
$q_{m}$
: Maximum adsorption capacity (mg g^−1^)
$K_{L}$
: Langmuir equilibrium constant (L mg^−1^)
$C_{e}$
: Equilibrium concentration
$\epsilon_{p}$
: Porosity
$D_{ef}$
: Effective diffusivity
$C_{s}$
: Surface concentration (mg L^−1^)
$C_{t}$
: Bulk concentration at time t (mg L^−1^)
$C_{o}$
: Initial concentration
$K_{f}$
: Film diffusion (cm s^−1^)
$D_{M}$
: Molecular diffusion
τ
: Tortuosity
$q_{ei,cal}$
: Predicted adsorption
${-r}_{A}$
: Reaction rate
$K_{1}$
: Constant rate (min^−1^)
$K_{2}$
: Constant adsorption (L mg^−1^ )
$C_{A}$
: Concentration at time t (mg L^−1^)
R
: Particle radius (nm)
$V_{m}$
: Molecular volume
$\rho_{p}$
: Particle density (kg m^−3^)
t
: Time (min)
M
: Molecular weight
$C_{t}$
: Bulk concentration at time t (mg L^−1^)
Φ
: Constant for water=2.27
$q_{ei,exp}$
: Experimental adsorption
n
: Number of experimental points

## Conflict of Interests

The authors declare no conflict of interest.

1

## Data Availability

The data that support the findings of this study are available from the corresponding author upon reasonable request.

## References

[1] Y. Boyjoo , M. Ang , V. Pareek , Chem. Eng. Sci. 2013,101, 764–784.

[2] H. Amiri , B. Ayati , H. Ganjidoust , Chem. Eng. Process 2017, 116, 48–59.

[3] G. Balasubramanian , D. D. Dionysiou , M. T. Suidan , I. Baudin , J. M. Laıné , Appl. Catal. B 2004, 47(2), 73–84.

[4] R. J. Braham , A. T. Harris , Ind. Eng. Chem. Res. 2009, 48(19), 8890–8905.

[5] B. Stephan , L. Ludovic , W. Dominique , Chem. Eng. J. 2011, 169, 216–225.

[6] H. Freudenhammer , D. Bahnemann , L. Bousselmi , S. U. Geissen , A. Ghrabi , F. Saleh A Vogelpohl , Water Sci. Technol. 1997, 35(4), 149–156.

[7] E. Baniasadi, G. F. Naterer, Springer., New York, **2013**, pp.461–491.

[8] J. R. Welty, Fundamentos de transferencia de momento, calor y masa, Limusa, México, **1994**.

[9] J. M. Smith, Ingeniería de la Cinética Química, Compañía Editorial Continental, México, **1991**.

[10] L. Rimoldi , D. Meroni , E. Falletta , A. M. Ferretti , A. Gervasini , G. Cappelletti , S. Ardizzone , Appl. Surf. Sci. 2017, 424, 198–205.

[11] M. Thommes , K. Kaneko , A. V. Neimark , J. P. Olivier , F. Rodriguez , J. Rouquerol , K. S. Sing , Pure Appl. Chem. 2015, 87, 1051–1069.

[12] S. Yurdakal, C. Garlisi, L. Özcan, M. Bellardita, G. Palmisano, Photocatalyst Characterization Techniques: Adsorption Isotherms and BET, SEM, FTIR, UV–Vis, Photoluminescence, and Electrochemical Characterizations, in: Heterogeneous Photocatalysis: Relationships with Heterogeneous Catalysis and Perspectives, **2019**, 87–152.

[13] A. Y. Shan , T. I. M. Ghazi , S. A. Rahid , Appl. Catal. A 2010, 389(1), 1–8.

[14] M. Kinoshita , Y. Shimoyama , J. Supercrit. Fluids. 2018, 138, 29–35.

[15] R. S. Dubey , K. V. Krishnamurthy , S. Singh , Results Phys. 2019, 14, 102390.

[16] B. González , B. Muñoz , M. A. Vicente , R. Trujillano , V. Rives , A. Gil , S. Korili , Chem. Eng. 2018, 2(2), 1–13.

[17] B. González , R. Trujillano , M. A. Vicente , V. Rives , S. A. Korili , A. Gil , Appl. Clay Sci. 2019, 167, 43–49.

[18] D. Papoulis , S. Komarneni , D. Panagiotaras , E. Stathatos , K. C. Christoforidis , M. Fernández , H. Li , Y. Shu , T. Sato , H. Katsuki , Appl. Catal. B. 2014, 147, 526–533.

[19] H. G. Charles , D. Smith , A. Huitson , J. Colloid Interface Sci. 1974, 47(3), 755–765.

[20] S. Bekkouche , M. Bouhelassa , N. Hadj Salah , F. Z. Meghlaoui , Desalination. 2004, 166, 355–362.

[21] N. Deedar , A. Irfan , A. Q. Ishtiaq , J. Environ. Sci. 2009, 21(3), 402–408.

[22] H. Freudenhammer , D. Bahnemann , L. Bousselmi , S. U. Geissen , A. Ghrabi , F. Saleh , A. Vogelpohl , Water Sci. Technol. 1997, 35(4), 149–156.

[23] X. Li , K. Peng , H. Chen , Z. Wang , Sci. Rep. 2018, 8(1), 1–11. 30076318 10.1038/s41598-018-29563-8 PMC6076233

[24] J. Ranogajec , M. Radeka , Z. Bačkalić , A. Škapin , D. Zorić , Chem. Ind. Chem. Eng. Q. 2010, 16(2), 117–126.

[25] A. Sraw , T. Kaur , Y. Pandey , A. Sobti , R. K. Wanchoo , A. P. Toor , J. Environ. Chem. Eng. 2018. 6(6), 7035–7043.

[26] C. Guillard , J. Disdier , C. Monnet , J. Dussaud , S. Malato , J. Blanco , J. M. Herrmann , Appl. Catal. B 2003, 46(2), 319–332.

[27] S. Alijani , M. Vaez , M. A. Zarringhalam Moghaddam , JChPE 2019, 53(1), 37–51.

[28] D. Lin-Vien, N. B. Colthup, W. G. Fateley, J. G. Grasselli, The handbook of infrared and Raman Characteristic Frequencies of Organic Molecules, Elsevier, **1991**.

[29] S. Duraipandian , M. M. Knopp , M. R. Pollard , H. Kerdoncuff , J. C. Petersen , A. Müllertz , Anal. Methods. 2018,10(29), 3589–3593.

[30] V. Ponnusami , K. S. Rajan , S. N. Srivastava , Chem. Eng. J. 2010, 163(3), 236–242.

[31] R. d. F. P. M. Moreira , T. P. Sauer , L. Casaril , E. Humeres , J. Appl. Electrochem. 2008, 35, 821–829.

[32] C. A. Aguilar , A. De la Cruz , C. Montalvo , A. Ruiz , S. Oros , S. Figueroa , M. Abatal , F. Anguebes , V. Cordova , Front. Environ. Sci. 2022, 10, 1–11.

[33] McKay , S. McKee , H. R. J. Walters , Chem. Eng. Sci. 1987, 42(5), 1145–1151.

[34] K. H. C. Keith , J. F. Porter , M. Gordon , Chem. Eng. Sci. 2004, 59(3), 501–512.

